# Research Progress on the Regulation and Developmental Utilization of Bioactive Metabolites Synthesis in *Floccularia luteovirens*

**DOI:** 10.3390/jof11120854

**Published:** 2025-11-30

**Authors:** Tongjia Shi, Lihua Tang, Siyuan Gou, Wensheng Li, Chunxiao Xu, Xu Zhao

**Affiliations:** 1 Institute of Urban Agriculture, Chinese Academy of Agricultural Sciences, Chengdu 610299, China; 2 Chengdu National Agricultural Science and Technology Center, Chengdu 610299, China; 3 The Edible Fungi Research Institute, Shanghai Academy of Agricultural Sciences, Shanghai 201403, China; 4 College of Food and Biological Engineering, Chengdu University, Chengdu 610106, China

**Keywords:** *Floccularia luteovirens*, metabolites, environmental regulation, multi-omics, liquid-state fermentation, bioactivity

## Abstract

*Floccularia luteovirens* is a rare edible and medicinal fungus endemic to the Qinghai–Tibet Plateau, prized for its abundance of high-value bioactive metabolites such as polysaccharides, terpenoids, and ergothioneine, which exhibit a variety of biological activities including immunomodulatory, antioxidant, and antitumor effects. Due to the current lack of successful domestication and limited wild resources, liquid fermentation technology has become an important strategy for the large-scale production of its mycelium and bioactive components. This review systematically summarizes the biological characteristics of *F. luteovirens*, the diversity of its metabolites, biosynthetic pathways, regulatory mechanisms influenced by environmental factors, and the application of multi-omics technologies in related research. It is suggested that future studies should integrate multi-omics approaches to elucidate its stress response and metabolic regulatory networks, and achieve high-value utilization of this resource through stress-resistant breeding and optimization of fermentation processes.

## 1. Introduction

Rare edible and medicinal fungi represent a significant component of the edible mushroom industry, possessing considerable economic value and serving as a key focus of scientific research. Globally, approximately 150,000 fungal species have been identified, of which over 2000 are edible and widely distributed across Asia, Europe, the Americas, Africa, and Oceania [1,2]. To date, more than 100 species of edible and medicinal fungi have been successfully cultivated artificially. According to FAO statistics from 2018, the global annual production of mushrooms has reached tens of millions of tons, with Asia remaining the dominant producing region [3]. China, with its vast territory and rich fungal resources, has seen an increasing number of rare edible and medicinal species identified and developed in recent years, driven by technological progress, indicating promising industrial prospects. Among these, *Floccularia luteovirens* (Alb. & Schwein.) Pouzar, a rare edible and medicinal fungus endemic to the alpine meadows of the Qinghai–Tibet Plateau, is of particular research and development value due to its abundance of bioactive compounds such as polysaccharides, terpenoids, and flavonoids [4].

However, the utilization of *F. luteovirens* faces multiple challenges. The species has not yet been successfully domesticated, and reliance on limited wild resources is insufficient to meet demand [5]. From a scientific perspective, although existing studies have revealed the potential of certain bioactive components (e.g., polysaccharides and terpenoids) in immunomodulation, antibacterial, antioxidant, and antitumor activities in vitro [6,7], there remains a lack of systematic in vivo functional validation, metabolic pathway analysis, and detailed interpretation of structure–activity relationships [8]. Moreover, while liquid fermentation is regarded as an effective approach for the large-scale production of mycelium and active metabolites (such as proteins, polysaccharides, and terpenoids) [9], issues such as low target metabolite yields and unclear metabolic regulation mechanisms still hinder its efficient development. Therefore, it is imperative to strengthen fundamental biological studies to decipher the molecular regulatory networks underlying its growth and secondary metabolism. Concurrently, liquid fermentation processes should be optimized, and metabolic engineering strategies should be employed to enhance the production of target compounds. Systematic pharmacological evaluation and safety assessments are also needed to facilitate the translation of laboratory findings into functional products.

To address these challenges, this review systematically outlines the research trajectory of *F. luteovirens*, from its ecological distribution to comprehensive development, focusing on the following aspects: assessing ecological characteristics and resource status, employing multi-omics analyses to reveal its secondary metabolic potential, elucidating the biosynthetic pathways and regulatory mechanisms of key active ingredients, and guiding the optimization of fermentation and extraction processes—including nutrient and environmental parameter modulation, fermentation process intensification, and improvements in extraction and drying strategies. Ultimately, this work aims to promote the application of *F. luteovirens* in functional foods and drug lead compounds, providing a reference for the high-value development of rare edible fungi. Therefore, this review aims to systematically consolidate the current knowledge on the biosynthesis, regulation, and utilization of metabolites in *F*. *luteovirens* to bridge the gap between its fundamental biology and industrial application. Specifically, we seek to (1) summarize the diversity and bioactivities of key metabolites, such as polysaccharides and terpenoids; (2) elucidate their biosynthetic pathways and the molecular and environmental mechanisms regulating their production; (3) evaluate advances and challenges in artificial cultivation and liquid fermentation for scalable production; and (4) identify critical research gaps and propose future directions for its high-value exploitation. By integrating research from ecological distribution to process optimization, this work provides a comprehensive framework to advance the use of *F. luteovirens* in functional foods and pharmaceutical leads.

## 2. Methodology

### 2.1. Search Strategies and Number of References

To ensure the systematicity, transparency, and reproducibility of this review, a rigorous literature search and screening process was implemented. The specific strategies and criteria are detailed below.

### 2.2. Screening Criteria and Process

The literature screening followed a structured process to minimize bias: (1) Initial Screening: Duplicate articles removed using reference management software—Zotero 7.0.30. (2) Title and Abstract Screening: The remaining articles were screened based on their titles and abstracts against the inclusion and exclusion criteria. Irrelevant studies were excluded at this stage. (3) Full-Text Assessment: The full texts of the potentially relevant articles were retrieved and thoroughly evaluated to determine their final eligibility.

### 2.3. Search the Database

The search was performed in the following electronic databases to ensure comprehensive coverage: Web of Science core collection, PubMed, Science Direct, CNKI (China National Knowledge Infrastructure), Wanfang Data.

### 2.4. Search Keywords

The search strategy employed a combination of keywords and their synonyms related to the organism and its metabolites. Key English terms included: “*Floccularia luteovirens*”, “*Armillaria luteo-virens*” (Former name), “yellow mushroom”, “polysaccharide”, “terpenoid”, “biosynthesis”, “fermentation”, “metabolite”, and “genome”. Corresponding Chinese keywords (e.g., “黄绿卷毛菇”, “黄绿蜜环菌”, “多糖”, “萜类”, “发酵”) were used for Chinese databases. Boolean operators (AND, OR) were applied to combine these terms effectively.

### 2.5. Time Range

The literature search covered the period from the inception of each respective database up to September 2025, to include both foundational historical research and the most recent advances in the field.

## 3. Biological Characteristics and Ecological Adaptations of *F. luteovirens*

### 3.1. Geographical Distribution and Habitat

*F. luteovirens* is a valuable edible fungus endemic to high-altitude areas in China, with its core geographical distribution centered on the Qinghai–Tibet Plateau and its surrounding areas. This range specifically encompasses Qinghai Province, Tibet Autonomous Region, and the Garzê Tibetan Autonomous Prefecture in Sichuan Province, spanning latitudes from 29°33′ to 38°09′ N and longitudes from 90°04′ to 102°01′ E [10]. In terms of its habitat, this species relies on high-altitude grassland and alpine meadow ecosystems, with a vertical distribution range of 3200~4800 m, and the unique plateau soil texture and climatic conditions together constitute its ideal habitat [11]. Specific climatic parameters show that in terms of temperature, it is highly adaptable and can tolerate the cold environment of the plateau, with the average temperature of the coldest month (January) ranges from −17.4–3.8 °C, and the average temperature of the summer and autumn seasons (June to September), the average temperature is between 6.2 °C and 15.9 °C. Regarding humidity, it experiences distinct moisture conditions: the mean relative humidity during the growing season is 41% to 74%, annual precipitation ranges from 344 to 574 mm, mean annual evaporation is 1393.8 to 2441.4 mm, and the humidity coefficient ranges from 0.42 to 0.78 [10]. These moisture characteristics, defined by “low precipitation and high evaporation” align well with the arid high-altitude environment.

### 3.2. Adaptation Mechanisms

#### 3.2.1. Environmental Adaptability of Macro Growth Cycle

The habitat specificity of high-altitude grassland on the Tibetan Plateau imposes compound extreme environmental pressure on *F. luteovirens*: in addition to strong ultraviolet radiation, it is subject to multiple limiting factors such as low-temperature stress, sharp diurnal temperature differences (up to 15–20 °C), and low soil organic matter content (resulting in nutrient-poor conditions), with synergies existing between these factors. This multifaceted stress regime acts as a potent selective filter across its entire life cycle, from mycelial colonization and primordium initiation to fruiting body maturation. In response, *F. luteovirens* has evolved a suite of targeted adaptive strategies, integrating eco-physiological adjustments in growth phenology with molecular-level mechanisms—such as enhanced antioxidant defense and stress-responsive gene expression—to successfully colonize this challenging ecological niche.

The growth cycle of *F. luteovirens* exhibits strict seasonality and environmental dependence: fruiting bodies appear only briefly from late summer to early autumn (June to September), a period that coincides with rising temperatures on the plateau and relatively concentrated rainfall. Its growth process is jointly regulated by soil temperature and humidity—only when soil temperature stabilizes between 6 and 16 °C and humidity remains at 40–70% can the mycelium rapidly expand and differentiate to form fruiting bodies. As temperatures drop (after October), the fungus enters dormancy, shortening its growth window to avoid unfavorable conditions [12].

#### 3.2.2. Adaptation Mechanisms at the Molecular Level

Currently, multi-omics technologies—including genome, transcriptome, metabolome, proteome analyses—have become an important tool to analyze the adaptive mechanisms of biological environments—by analyzing multi-dimensional genomics data under droughts, low temperatures, salinity and alkalinity, the adaptive strategies of organisms in the areas of gene expression regulation, metabolic network remodeling and functional protein synthesis can be systematically revealed [13,14,15,16]. As summarized in Table 1, recent molecular omics studies have advanced our understanding of environmental adaptation in *F. luteovirens*. However, research on its molecular mechanisms underlying environmental adaptation remains preliminary. Current studies have predominantly focused on transcriptomic responses to single-factor UV stress, leading to the identification of several differentially expressed genes associated with pigment synthesis and antioxidant responses—such as those involved in riboflavin biosynthesis [17]. Yet these findings fail to capture the integrated regulatory network activated under the combined stresses of low temperature, high UV radiation, and nutrient deficiency that characterize its natural alpine habitat. Furthermore, metabolic characteristics across different developmental stages—mycelial, primordium, and fruiting body phases—and their dynamic coupling with environmental factors remain to be fully elucidated.

A key area warranting in-depth exploration is whether the adaptation of *F. luteovirens* to plateau extremes relies on an integrated signal perception and transduction system, potentially centered on the crosstalk and amplification of multiple stress signals at the second messenger level. One critical scientific question arises: under combined stress conditions, does *F. luteovirens* employ a signaling hub composed of secondary messengers such as ROS, Ca^2+^, and NO—similar to the mechanism identified in *Ganoderma* species as described in Section 4.2.2? If so, how does this network dynamically integrate upstream signals—UV radiation, low temperature, and nutrient scarcity—to coordinately regulate downstream physiological processes including pigment synthesis, antioxidant defense, and energy metabolism? A systematic dissection of these questions will provide a comprehensive understanding of the alpine adaptation mechanisms of *F. luteovirens* from an integrative biology perspective, and establish a theoretical foundation for its artificial domestication and targeted metabolic regulation.

## 4. Bioactive Metabolites of *F. luteovirens*

### 4.1. Diversity of Bioactive Metabolites

#### 4.1.1. Metabolome Characteristics and Product Diversity of Mycelium

*F. luteovirens* is rich in proteins, amino acids, sugars, alkaloids, and small amounts of organic acids, flavonoids, cardiac glycosides, steroidal triterpenoids, glycosides, and saponins [25]. In edible fungi research, the application of metabolomics helps to systematically analyze macrofungal metabolites, elucidate metabolic patterns [26], screen bioactive substances, and optimize cultivation conditions [27], offering important technological support for the in-depth exploration of the metabolic potential of mycelium. However, metabolomic studies on *F. luteovirens* are mainly limited to comparative analyses of the composition of wild fruiting bodies, while the metabolite profiles of the mycelial stage remain less studied. As metabolites in the mycelium serve as the basis for the synthesis and accumulation of bioactive substances, and given that metabolomics, as a comprehensive analytical method, is capable of systematically analyzing low-molecular-weight metabolites in organisms, it directly reflecting metabolic changes under different conditions [28]. The study by Liu et al. provided a 27 Mb reference-level genome of *F. luteovirens*, containing 7068 protein-coding genes, and predicted the genome composition and gene functions [29]. Genome enrichment and pathway analysis indicated the potential biosynthesis of terpenoids, polyketides, and polysaccharides in *F. luteovirens*. Previous studies have analyzed the enzyme kinetics and metabolic profiling of the liquid culture system of *F. luteovirens*; untargeted metabolomics identified more than 3569 metabolites, including 148 terpenoids, 26 alkaloids, and 31 flavonoids in liquid cultured mycelia over a 20-day culture cycle, with differential metabolites focusing mainly on carboxylic acid derivatives and lipids [4].

#### 4.1.2. Key Signature Metabolites

**Figure 1:**
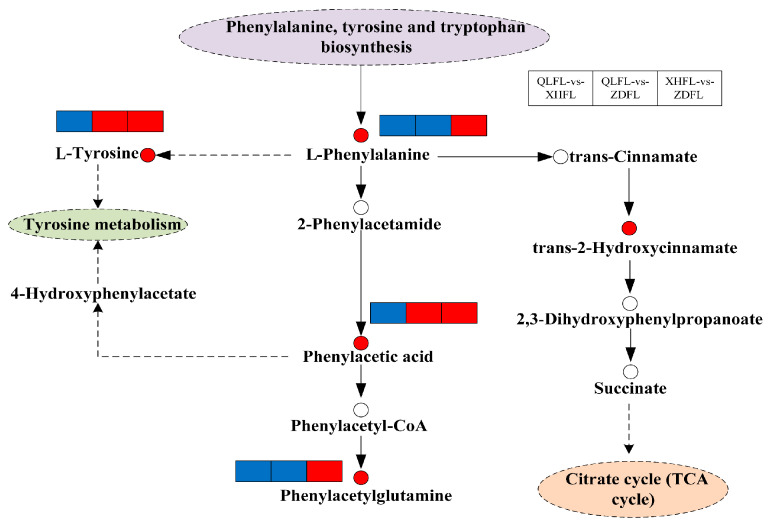
Overview of metabolic pathways mapped to possible regulation of key metabolites in pairwise comparisons of *F. luteovirens* from different regions. Note: Small red circles indicate significant upregulation of metabolites; small empty circles indicate undetected metabolites; small red rectangles indicate significant upregulation of metabolites between groups; small blue rectangles indicate significant downregulation of metabolites between groups; solid arrows represent facilitation, and dotted arrows represent indirect facilitation (Tang et al., 2024 [22]).

Recent studies have shown that geographic differences in the antioxidant activity of *F. luteovirens* are mainly associated with the regulation of its phenylalanine biosynthesis and metabolic pathways. Targeted metabolomics analysis revealed that vanillic acid may be a key metabolite affecting its antioxidant capacity [22]. Further results showed that vanillic acid content varied significantly among samples from different geographical sources and exhibited a significant positive correlation with total flavonoid content, glutathione level, and hydroxyl radical scavenging capacity (Figure 1). Consequently, vanillic acid was identified as a key marker for distinguishing antioxidant activity. On the other hand, the unique yellow phenotype of this species was mainly attributed to the accumulation of riboflavin, whose content directly determines the color expression of the fruiting bodies, and the expression of the riboflavin transporter protein gene *FIMCH5* was closely associated with the formation of this phenotype [20]. Based on the above findings, vanillic acid and riboflavin can be used as important chemical markers for origin identification, authenticity recognition, and quality assessment of *F. luteovirens*. The riboflavin content is utilized for morphology-assisted identification, while the vanillic acid level is used for an effective assessment of antioxidant quality.

### 4.2. Biological Function of Key Active Ingredients

#### 4.2.1. Polysaccharides

Current research on the key bioactive components of *F. luteovirens* is summarized in Table 2. The polysaccharides exhibit remarkable antioxidant and antitumor activities, along with hygroscopic and moisturizing properties, demonstrating their broad application potential in health foods and cosmetics [30,31,32]. Specifically, the *F. luteovirens* polysaccharide (FLP1) from the fruiting bodies significantly enhances intestinal immune responses via the MAPK/Nrf2 signaling pathway, improves gut microbiota structure, and effectively alleviates immunosuppression [33,34]. It is important to note, however, that these compelling findings are predominantly derived from mouse models. Their relevance to human physiology remains to be established, and the exact structural motifs of FLP1 responsible for these effects are yet to be conclusively identified. Multiple studies have shown that both *F. luteovirens* polysaccharides (FLPs) and exopolysaccharides (FLEP) display significant scavenging capacities against DPPH and ABTS free radicals, inhibit cancer cell proliferation in vitro, and exert protective effects against hydrogen peroxide-induced oxidative damage in cell models [31,35]. While these results are promising, the direct correlation between simple chemical antioxidant assays and complex in vivo health benefits is often weak, representing a common challenge in the field of functional food research. Furthermore, owing to their excellent moisture absorption and retention properties, FLPs show promising cosmetic applications. Notably, the exopolysaccharide exhibits both antibacterial and tyrosinase inhibitory activities, making it suitable for preserving aquatic products such as Pacific white shrimp and significantly extending their shelf life [32,36]. Recent studies also indicate that edible coatings formulated with FLPs and chitosan can be effectively applied in the preservation of aquatic products like tuna [37].

#### 4.2.2. Terpenoids

Terpenoids represent a major class of bioactive natural products in fungi, classified based on the number of isoprene units into monoterpenes (C10), sesquiterpenes (C15), diterpenes (C20), sesterterpenes (C25), triterpenes (C30), and tetraterpenes (C40). Their biosynthesis is initiated from the universal precursors isopentenyl pyrophosphate (IPP) and dimethylallyl pyrophosphate (DMAPP) [44,45]. Fungi serve as a prominent source of diverse terpenoids. Advances in sequencing technologies and bioinformatics have established genome mining as one of the most powerful strategies for discovering novel terpenoids from fungal genomes [46]. To date, numerous terpene cyclases have been identified, including canonical class I and class II terpene cyclases, emerging UbiA-type membrane-bound terpene cyclases, as well as various tailoring enzymes such as cytochrome P450 monooxygenases, flavin-dependent monooxygenases, and acyltransferases [47]. Among terpenoids, triterpenes are predominantly distributed in certain basidiomycetes. Lanosterol serves as the key biosynthetic precursor for ergosterol and other triterpenoid metabolites, which exhibit a range of pharmacological activities including antitumor and immunomodulatory effects [38]. Meanwhile, sesquiterpenoids also demonstrate notable bioactivities. For instance, protoilludane-type sesquiterpene aryl esters extracted from *F. luteovirens* showed significantly improved yield following process optimization. These compounds exhibit remarkable antioxidant activity, with DPPH and hydroxyl radical scavenging rates reaching (62.60 ± 1.88)% and (95.99 ± 1.74)%, respectively. Furthermore, they displayed potent inhibitory effects against Escherichia coli, Staphylococcus aureus, and Bacillus cereus, highlighting their potential as natural food antioxidants and preservatives [39]. However, the evidence is currently limited to in vitro assays. Future research must address critical aspects such as efficacy in real food matrices, sensory impact, and in vivo safety to substantiate their practical application.

#### 4.2.3. Other High-Value Compounds

Studies have shown that *F. luteovirens* is rich in a variety of highly bioactive compounds, exhibiting significant development potential. Using hydrophilic interaction chromatography (HILIC) coupled with online HPLC-DPPH activity screening, high-purity L-(+)-ergothioneine was efficiently isolated from its methanol extract. This compound is a rare amino acid-derived antioxidant, with a content as high as 5.36 mg/g in the fruiting bodies. It can accumulate within cells via specific transporters, efficiently scavenge free radicals, and is hailed as the “longevity vitamin,” making it an excellent source for functional foods and health products [40]. Furthermore, the low molecular weight fraction (<6 kDa) obtained by hollow fiber membrane fractionation significantly induced apoptosis in non-small cell lung cancer cells by activating the Caspase-3 pathway. The active constituents include various amino acids, nucleosides, terpenoids, and alkaloids, providing a new direction for anti-tumor drug development [41]. Simultaneously, the *F. luteovirens* water extract (FLW), as a multi-component complex system, demonstrated comprehensive efficacy in a type II diabetes model by activating the Nrf2/HO-1 pathway and inhibiting the mTOR/GSK-3β pathway, thereby improving glucose and lipid metabolism, and alleviating oxidative stress and inflammatory responses. In a migraine model, it also modulated neurotransmitter levels, effectively alleviating pain and inflammation, showcasing multi-target regulatory therapeutic characteristics and broad application prospects in the prevention and adjuvant treatment of metabolic and neurological diseases [35,42]. Additionally, lipids extracted via supercritical CO_2_ extraction were analyzed by GC-MS, identifying 25 fatty acids, including linoleic acid (relative content 10.6%) and other unsaturated fatty acids, further clarifying the material basis for its nutritional and functional components [43].

### 4.3. Major Biosynthetic Pathways

#### 4.3.1. Polysaccharide Biosynthetic Pathway

Research on the polysaccharide structure of *F. luteovirens* remains at an early stage. Liu et al. isolated and purified a major polysaccharide fraction, GLP, using Sepharose CL-6B gel column chromatography [48]. Structural characterization by infrared spectroscopy and nuclear magnetic resonance (NMR) revealed that GLP is an arabinoxylan composed of α-D-xylopyranose and β-D-arabinopyranose residues, featuring a backbone of 1 → 4 linkages with 1 → 6 branched chains. In another study, Liu Yan characterized the molecular structures of ALP-1, ALP-2, and ALP-3 using an integrated approach combining partial degradation, methylation analysis, NMR spectroscopy, and FT-IR [30]. All three polysaccharides were identified as glucans. ALP-1 primarily contains T-, 1,3-, and 1,3,6-linked glucopyranosyl residues, representing a β-1,3-glucan backbone branched at the C6 position. In contrast, ALP-2 and ALP-3 are characterized by α-1,4-linked backbones, classifying them as glycogen-like or amylose-like glucans.

Our group has elucidated the dynamic process of polysaccharide biosynthesis in *F. luteovirens* at the physiological level by analyzing enzyme activities and metabolite profiles across different culture periods. Polysaccharide production peaked on day 20, with intracellular polysaccharides reaching 23.78 ± 0.90 mg/g and extracellular polysaccharides at 0.93 ± 0.11 mg/mL. This period also coincided with maximal activities of hydrolytic enzymes such as amylase and cellulase, which degrade macromolecular carbon sources into small sugars, thereby supplying essential precursors like UDP-glucose and UDP-xylose for polysaccharide assembly [4]. Metabolomic analysis further indicated continuous accumulation of amino acids—including L-glutamine, L-histidine, L-tyrosine, and L-proline—throughout the cultivation, with significant enrichment of metabolic pathways such as alanine, aspartate, and glutamate metabolism. This active amino acid metabolism not only furnishes energy (ATP) and reducing equivalents (NADPH) but also provides amino donors—particularly glutamine—for the biosynthesis of amino sugars (e.g., glucosamine, a component of chitin and certain polysaccharides) [49].

Ultimately, polysaccharide biosynthesis is tightly regulated at the genetic level. Although no gene clusters directly involved in polysaccharide synthesis have been reported in *F. luteovirens*, studies on other edible macro-fungi suggest that the synthesis of different polysaccharide types relies on specific glycosyltransferase (GT) gene families [50,51]. The expression of these genes is finely tuned by cultural conditions, nutrient availability, and metabolic cues—including the aforementioned amino acids—thereby governing the efficiency, chain length, and branching pattern of polysaccharide synthesis. We thus hypothesize that supplementing the culture medium with specific amino acids may not only boost polysaccharide yield by supplying precursors and energy but also modulate the expression of key biosynthetic genes via upstream signaling pathways, leading to more efficient polysaccharide production. Future work should focus on identifying these key genes and elucidating their regulatory mechanisms, thereby establishing a foundation for the targeted synthetic biological engineering of FLPs.

#### 4.3.2. Triterpenoid Biosynthetic Pathways

Fungi within the Ascomycota and Basidiomycota phyla are prolific producers of structurally diverse terpenoids. While the biosynthesis of several sesquiterpenoid mycotoxins and bioactive diterpenoids in ascomycetes has been extensively characterized, the molecular mechanisms underlying terpenoid biosynthesis in basidiomycetes—including many macrofungi—remain largely unexplored, despite their capacity to synthesize a wide array of terpenoid natural products [52].

Among metabolites, triterpenoids represent core pharmacologically active constituents, and their biosynthetic pathways along with associated environmental regulation are increasingly becoming a research focus. The mevalonate (MVA) pathway is widely recognized as the primary route for triterpenoid biosynthesis: acetyl-CoA is sequentially converted by a series of enzymes, including the rate-limiting enzyme 3-hydroxy-3-methylglutaryl-CoA reductase (HMGR), to farnesyl pyrophosphate (FPP) [41]. FPP is then processed by squalene synthase (SQS) and squalene epoxidase (SE), culminating in cyclization by lanosterol synthase (LS) to form the triterpenoid skeleton. Subsequent modifications by cytochrome P450 monooxygenases contribute to the structural diversity of triterpenoids [53,54]. HMGR serves as the key rate-limiting enzyme governing the efficiency of this pathway.

Advances in genomics have enabled the use of bioinformatics tools to mine biosynthetic gene clusters (BGCs), providing a powerful approach to elucidate secondary metabolite biosynthesis [55]. For instance, in the basidiomycete *Hypholoma sublaterium*—a producer of the antitumor compound clavicoric acid—oxidosqualene synthase and squalene epoxidase genes have been identified as likely key players in its biosynthesis [56]. Genomic sequencing (27 Mb) and functional annotation of *F. luteovirens* by Liu et al. further revealed considerable potential for the synthesis of metabolites such as terpenoids, polyketides, and polysaccharides in this species [29].

Environmental regulation is increasingly regarded as an effective strategy for activating silent BGCs and optimizing the production of target metabolites. Studies have shown that variations in culture conditions—such as carbon and nitrogen sources, pH, and aeration—as well as stress factors including light, temperature, and reactive oxygen species, can markedly influence terpenoid output and profile [57,58,59,60,61]. These effects are often mediated through transcriptional regulation of key genes such as terpene synthases, across developmental stages including mycelia, primordia, and fruiting bodies. For example, exogenous methyl jasmonate (MeJA) was shown to enhance triterpenoid accumulation in *G. lucidum* and upregulate key triterpenoid biosynthetic genes (e.g., HMGR, FPS, LS). Coupled with gene overexpression and silencing techniques, several candidate genes involved in triterpenoid biosynthesis and regulation have been identified [62]. Omics analyses further revealed that MeJA induces metabolic reprogramming in *G. lucidum*, suppressing primary metabolic processes such as glucose metabolism and protein synthesis while favoring the production of secondary metabolites like triterpenoids [63]. Thus, rational optimization of environmental conditions offers a viable means to efficiently induce triterpenoid biosynthesis without the need for complex genetic engineering [52,64].

In summary, although progress has been made in identifying key enzymes, gene clusters, and environmental regulators of triterpenoid biosynthesis in basidiomycetes—with species such as *F. luteovirens* showing promising metabolic potential—the complete elucidation of terpenoid pathways, including BGC-based biosynthetic mechanisms and multi-level regulatory networks, remains an important goal. Future efforts should integrate multi-omics and synthetic biology strategies to advance our understanding of triterpenoid biosynthesis in basidiomycetes and provide a theoretical foundation for their efficient exploitation.

## 5. Genetic and Environmental Regulation of Bioactive Metabolites Synthesis in *F. luteovirens*

### 5.1. Molecular Basis and Genetic Regulation of Synthesis

Natural products have consistently served as a pivotal source for the discovery and development of therapeutics for human use, as well as products for animal health and crop protection [65,66,67]. Microbial genome mining emerged as an important strategy for revitalizing drug discovery in the early 2000s; at that time, researchers discovered that the number of secondary metabolite BGCs encoded in newly sequenced actinomycete genomes far exceeded the sizes predicted based on known metabolites [68,69]. In recent years, with the continuous advancement of genome mining technology, an increasing number of novel natural products have been identified and characterized [70]. Moreover, genome mining provides synthetic biology with a growing repository of genetic building blocks and tools. This advancement facilitates the design of novel bioactive secondary metabolite derivatives, such as antibiotics, through combinatorial biosynthesis strategies [71].

In this context, genome analysis of specific microorganisms has become an important way to exploit their medicinal potential. For example, a high-quality genome assembly of Ophiocordyceps sinensis revealed a genome size of approximately 110.8 Mb and 63 secondary metabolite gene clusters, including multiple polyketide synthases, non-ribosomal peptide synthetases, and others [72]. Similarly, genome sequencing of Morchella esculenta annotated a total of 9550 protein-coding genes and predicted the possible structures of its metabolites [73]. The value of this resource in natural product development was further highlighted by the genome study of *F. luteovirens*: its genomes was approximately 27–28.8 Mb, encoding 7000–8333 protein-coding genes, and phylogenetic analyses confirmed its belonging to the genus *Floccularia*. The study identified 400 Carbohydrate-Active enZyme (CAZyme) genes and 357 species-specific gene families, providing a genetic basis for its polysaccharide metabolism and formation of unique medicinal components [74]. More notably, the study also predicted 16 secondary metabolite BGCs, including clusters known to produce guadinomine with antimicrobial activity and melleolides with both entomotoxic and antimicrobial activity [29]. In addition, the genome encodes 400 CAZyme genes and 357 species-specific gene families [74], providing the molecular basis for the synthesis of complex polysaccharides and specific metabolites. The key transporter protein gene *FlMCH5* was specifically highly expressed in yellow mushrooms, and functional experiments confirmed that its overexpression in tobacco significantly increased riboflavin content (up to 36.94 μg/g) [20], suggesting that this gene plays a central regulatory role in riboflavin accumulation. Additionally, the study identified a variety of metabolites including 148 terpenes and phenolics, such as vanillic acid [4,22], further confirming the potential of this species in synthesizing high-value natural products such as riboflavin, terpenoids, and phenolic acids at the level of genetic regulation. These studies have systematically revealed the genetic basis and regulatory mechanism of secondary metabolite synthesis in *F. luteovirens*, highlighting its important value in the development of medicinal active ingredients.

A series of key functional genes and their regulatory mechanisms have been revealed in *F. luteovirens*. The specific high expression of the core transporter protein gene *FlMCH5* was shown to be the key genetic basis for riboflavin accumulation and the formation of the yellow phenotype of the fruiting bodies [20]. In addition, this species possesses a strong environmental stress response capacity, with 225 differentially expressed genes (DEGs) induced by strong UV radiation, which are significantly enriched in the pathways of environmental signal transduction, DNA damage repair, and pigment metabolism, underscoring its transcriptional regulatory potential to cope with extreme environments [17]. The reliability of these functional gene studies is supported by a stable system of internal reference genes—studies have shown that genes such as *H3*, *ACT*, *EF-Tu*, etc. among others, are stably expressed under a variety of abiotic stresses, such as salt, drought, oxidation, heat, extreme pH, cadmium stress, etc., thus providing key technical support for the precise quantification and functional analysis of the related gene expression [18].

### 5.2. Regulatory Role and Mechanisms of Environmental Factors

#### 5.2.1. Regulation by Abiotic Stresses

Beyond genetic background and culture medium composition, the growth environment constitutes a pivotal factor influencing the formation of fruiting bodies in edible and medicinal fungi, directly determining essential agronomic traits including morphology, yield, flavor profile, and stress resistance. Deciphering the regulatory mechanisms through which environmental factors—such as temperature, humidity, light intensity and quality, and carbon dioxide concentration—govern the growth and metabolism of edible and medicinal fungi is instrumental for optimizing cultivation environment management strategies, thereby enhancing both the yield and quality of germplasm resources [75].

Abiotic stress represents a crucial strategy for modulating secondary metabolism in fungi. In edible fungi, diverse physical, chemical, and nutritional signals can function as effective stress stimuli, significantly influencing the biosynthesis of target metabolites through activation of specific signaling pathways—such as calcium signaling and reactive oxygen species (ROS) signaling—and upregulation of key enzymatic genes. In *G. lucidum*, for instance, various individual chemical or biological elicitors—including small molecules like MeJA, salicylic acid, and acetic acid, as well as metal ions such as Na^+^ and Cu^2+^ [76,77,78,79]—physical treatments (e.g., heat stress, pH modulation) [80,81], and nutritional conditions (e.g., carbon source, nitrogen source, and their ratio) have been demonstrated to effectively upregulate genes involved in ganoderic acid (GA) biosynthesis [82,83]. These responses are mediated through multiple mechanisms, including the calcium/calcineurin signaling pathway and alterations in membrane fluidity, among others.

Specifically, regarding *F. luteovirens*, studies have indicated that fruiting bodies from different geographical origins exhibit substantial regional disparities in metabolite abundance, confirming the decisive role of geographic provenance—with its associated environmental and climatic conditions—in determining quality and bioactivity [84]. Our team’s previous research revealed that triterpenoid accumulation in liquid cultures of *F. luteovirens* reached its peak at 20 days, synchronizing with the maximal activities of enzymes such as amylase and superoxide dismutase (SOD) [4]. We hypothesize that triterpenoid biosynthesis is linked to nutrient utilization and cellular redox balance. This “enzyme activity–metabolite accumulation” relationship is a common phenomenon in secondary metabolism [58], analogous to mechanisms reported in various fungi, such as the upregulation of genes like HMGR under nitrogen limitation [53,58].

Regarding temperature effects, heat stress may influence triterpenoid synthesis by modifying membrane fluidity. For example, studies on other fungi have shown that heat stress increases membrane fluidity and upregulates the expression of genes including HMGR and SQS [85], suggesting that a comparable mechanism may operate in *F. luteovirens*. In terms of light regulation, research on medicinal fungi has demonstrated that white light irradiation—particularly a three-stage strategy (2 days of dark culture → 6 days under 0.94 W/m^2^ white light → subsequent exposure to 4.70 W/m^2^ white light)—significantly enhances triterpenoid production, an effect attributed to the redirected metabolic flux toward triterpenoid biosynthesis [61]. Meanwhile, blue light has also been shown to promote triterpenoid accumulation in certain fungal species [86]. These findings provide valuable references for investigating light regulation in *F. luteovirens*. However, related experiments have not yet been initiated, and there remains a notable lack of analysis regarding the effects of different light intensities and phase-specific combinations on triterpenoid synthesis in this species.

#### 5.2.2. Cross-Talk of Stress Signals

The regulation of fungal secondary metabolism by abiotic stress involves a complex intracellular signaling network, characterized by notable “cross-talk” among different stress signaling pathways. As a basidiomycete known for producing bioactive metabolites, some edible and medicinal fungi has emerged as a potential model system for investigating how environmental factors regulate metabolism in basidiomycetes [85]. For instance, heat stress activates both nitric oxide (NO) and calcium ion (Ca^2+^) signaling pathways. These pathways exhibit upstream-downstream relationships and engage in cross-talk to modulate the biosynthesis of GA [87]. Further mechanistic studies reveal that heat stress triggers Ca^2+^ signaling via the activation of specific phospholipid signaling pathways involving PI-4-P and PIP2 [88]. In contrast, under Cu^2+^ stress, signaling cross-talk is manifested as a bidirectional interaction between ROS and Ca^2+^: Cu^2+^ induces ROS accumulation, leading to elevated Ca^2+^ levels, which in turn feedback-regulate ROS production. This interplay forms a finely tuned feedback loop that coordinates mycelial growth and GA synthesis [89]. Collectively, these studies illustrate from multiple perspectives that the synergy and cross-talk among multiple second messengers—such as NO, Ca^2+^, ROS, and phospholipid molecules—constitute a central mechanism enabling precise regulation of secondary metabolism in fungi in response to environmental stresses.

## 6. Biotechnological Utilization: From Mycelial Production to Functional Products

### 6.1. Strategies for Enhanced Metabolite Production

#### 6.1.1. Fermentation Process Optimization

Given the challenges in the artificial cultivation of *F. luteovirens* fruiting bodies, biotechnology, particularly liquid fermentation, has emerged as a sustainable and efficient strategy for the large-scale production of its mycelium and bioactive metabolites. The production of polysaccharides and other metabolites can be significantly enhanced through precise optimization of fermentation conditions. Lessons from other fungi, such as *Ganoderma lucidum*, demonstrate the profound impact of carbon and nitrogen source manipulation. For instance, optimizing the carbon-to-nitrogen (C/N) ratio to 20:10 was crucial for maximizing both biomass and GA yield [83]. Advanced strategies, like the synergistic use of exogenous elicitors with a variable-temperature regime in certain edible fungi, such as *Agaricus bitorquis*, have boosted mycelial polysaccharide yield by over 65% [90], underscoring the efficacy of integrated physical and chemical induction. For *F. luteovirens*, research on fermentation optimization remains nascent but promising. Initial studies have employed orthogonal design to refine medium composition [91] and introduced novel approaches like microparticles and surfactants to enhance extracellular polysaccharide (FLEP) yield [32]. Previous research has identified culture duration as a critical factor, with polysaccharide accumulation peaking at a specific fermentation time, beyond which degradation may occur [4]. However, a significant knowledge gap exists regarding the systematic optimization of key parameters (e.g., C/N ratio, dissolved oxygen, specific elicitors) for triterpenoid production. Future research must leverage insights from regulatory mechanisms (Section 5.2) to design intelligent fermentation strategies that deliberately induce stress responses to activate silent BGCs and enhance the yield of high-value terpenoids.

#### 6.1.2. Artificial Culture and Condition Optimization

The foundation for cultivating *F. luteovirens* was laid by early studies on carbon and nitrogen source utilization [92]. Subsequent work has systematically explored nutritional and environmental factors, including pH, temperature, and light, using single-factor and orthogonal designs [93,94,95]. A detailed investigation into light quality (white, red, yellow, blue) found no significant promotive effect on mycelial growth compared to dark conditions [96]. While this suggests light is not a primary requirement for vegetative growth, it does not preclude its potential role, observed in other fungi like *Monascus purpureus* and *Hypsizygus marmoreus* [97,98], in regulating secondary metabolism. Therefore, future studies should shift focus from mycelial biomass to the specific effects of light quality, photoperiod, and their interaction with other factors on the synthesis of pigments (e.g., riboflavin) and other valuable metabolites.

### 6.2. Downstream Processing for Product Development

#### 6.2.1. Extraction Process Innovation

The objective of downstream processing is the efficient, economical, and green recovery of bioactive compounds. For *F. luteovirens*, extraction strategies must be tailored to the target metabolite’s properties. The classical hot-water extraction, optimized via Response Surface Methodology (RSM), is effective for fruiting body polysaccharides (FLPs) [99]. In contrast, lipophilic compounds like protoilludane-type sesquiterpene aryl esters are more efficiently extracted with organic solvents (e.g., ethyl acetate) assisted by ultrasound (UAE), which significantly improves yield and bioactivity [43]. A notable advancement is the integration of in situ extraction during fermentation, where adding microparticles and surfactants increased FLEP yield by 1.5-fold [32]. The future lies in the flexible combination and optimization of modern techniques (UAE, MAE, SFE) based on the polarity, scale, and economic constraints of the target product.

#### 6.2.2. Influence of Post-Treatment (Drying) Methods

The drying process is a critical determinant of final product quality, directly influencing the preservation of heat-sensitive metabolites. Consistent evidence from *F. luteovirens* confirms that vacuum freeze-drying (VFD) best preserves antioxidant activity, metabolite integrity, and microstructure [100]. Metabolomic studies on *F. luteovirens* further identify amino acid metabolism as a core pathway responding to drying stress. However, the high cost of VFD limits its industrial application. Although thermal drying techniques such as microwave drying are more practical, they may lead to structural collapse and the loss of bioactive metabolites [101]. Notably, the optimal drying strategy is compound-dependent. Thus, the choice of drying method should be guided by the properties of target metabolites, and research on cost-effective hybrid technologies (e.g., hybrid hot air–microwave vacuum drying) should be prioritized to facilitate industrial application.

### 6.3. Cultivation Mode Comparison and Scale-Up Potential

A comparative analysis clearly positions liquid fermentation as the most viable near-term strategy for utilizing *F. luteovirens*, particularly when contrasted with the ongoing challenges of fruiting body cultivation, which remains unsuccessful and relies on limited, ecologically unsustainable wild harvests. In contrast, liquid fermentation of the mycelium offers a compelling alternative, as it is nutritionally similar to the fruiting body [102] and serves as a rich source of diverse bioactivities, including immunomodulatory polysaccharides and antioxidant terpenoids [31]. The feasibility of scaling up this process to a 70 L fermenter, supported by established kinetic models for growth and substrate consumption, lays a solid foundation for industrial production [103]. The paramount advantages of liquid fermentation lie in its sustainability and controllability, providing a year-round, reliable supply of uniform biomass independent of seasonal and environmental constraints, while also facilitating easier extraction and achieving higher economic efficiency compared to processing solid fruiting bodies [32,104]. Therefore, future commercialization efforts should prioritize the refinement of liquid fermentation processes, even as research toward the breakthrough of fruiting body domestication retains its scientific importance.

## 7. Conclusions and Outlook

### 7.1. Conclusions

As a rare edible and medicinal fungus endemic to the Tibetan Plateau, *F. luteovirens* has gained significant attention in health industry development and basic research owing to its abundant bioactive metabolites and distinctive environmental adaptability. This review demonstrates that the integrated application of multi-omics technologies serves as a central driving force for deciphering its biological processes. Genomic studies have uncovered its compact genome architecture and abundant BGCs, providing a genetic basis for the biosynthesis of pharmacologically active compounds such as guadinomine and melleolides. Integrated transcriptomic and metabolomic analyses have successfully elucidated the key mechanisms underlying riboflavin accumulation and yellow pigment formation, while functional genomics has established a methodological foundation for precise gene expression profiling under diverse stress conditions.

Notably, aiming to address the core scientific questions raised earlier regarding its alpine adaptation mechanisms, current research has yielded preliminary but critical insights: First, regarding whether *F. luteovirens* relies on an integrated signal perception and transduction system to adapt to plateau extremes, genomic annotation has identified 205 environmental adaptation genes, and transcriptomic analysis under UV stress has detected 225 DEGs significantly enriched in pathways of environmental signal transduction, DNA damage repair, and pigment metabolism—collectively confirming the existence of a multi-dimensional integrated signal perception and transduction system that provides a genetic basis for coping with complex extreme environments. Second, for the question of whether a signaling hub composed of secondary messengers (e.g., ROS, Ca^2+^, NO) exists under combined stress, metabolomic studies have identified vanillic acid as a key antioxidant marker, and the dynamic correlation between SOD) activity and triterpenoid accumulation further supports the core role of ROS in stress response. Combined with its synergistic adaptation to low temperature, high UV radiation, and nutrient scarcity, it is reasonable to infer that *F. luteovirens* may employ a secondary messenger crosstalk mechanism to integrate stress signals, though the specific molecular interaction network requires further experimental validation. Third, in terms of how upstream signals regulate downstream physiological processes, multi-omics data show that the specific high expression of the riboflavin transporter gene *FlMCH5* mediates pigment synthesis (yellow phenotype formation); the phenylalanine biosynthesis and metabolism pathway promotes vanillic acid accumulation to enhance antioxidant defense; and the synchronous dynamic changes in amylase/cellulase activity and polysaccharide synthesis optimize energy metabolism—forming a regulatory chain of “stress signal → gene expression → metabolic reprogramming → physiological adaptation”.

Overall, current research has preliminarily outlined the diversity of metabolites, bioactivities, and response patterns to environmental factors in *F. luteovirens*, affirming its considerable potential for applications in functional foods, pharmaceuticals, and cosmetics. Nevertheless, this field still confronts serious challenges: First, fundamental biological questions remain unresolved—artificial domestication has not yet been achieved, and the precise regulatory network governing secondary metabolism, particularly the signal integration mechanisms in response to combined stresses, remains poorly understood. Second, technical bottlenecks are prominent—large-scale production strategies such as liquid fermentation still face obstacles such as low yields of active products and unclear regulatory mechanisms. Third, research depth is inadequate—studies on the chemical structures, structure-activity relationships, and in vivo mechanisms of action of bioactive components remain relatively limited, resulting in insufficient deep processing of derived products and limited value addition.

### 7.2. Prospects

Future research on *F. luteovirens* should aim to achieve breakthroughs spanning mechanistic understanding, technological innovation, and industrial application. At the fundamental research level, it is essential to further deepen integrated multi-omics analyses, with a focus on deciphering the regulatory networks linking composite stress signals—such as low temperature, high solar radiation, and low oxygen—with secondary metabolite synthesis, taking into account habitat conditions. Attention should also be given to the role of epigenetic regulation, such as DNA methylation and histone modifications, in these processes. Concurrently, systematic comparisons of metabolic profiles across different developmental stages—including mycelia, primordia, and fruiting bodies—should be conducted.

At the technological development level, efforts should be intensified to promote the application of modern biotechnological tools. This includes leveraging gene editing technologies such as CRISPR-Cas9 in combination with conventional mutagenesis approaches for targeted breeding of stress-tolerant and high-yielding strains. Based on an in-depth understanding of the regulatory principles governing environmental factors, precision fermentation parameters—such as carbon and nitrogen sources, light quality, and temperature strategies—should be optimized. Additionally, innovative, efficient, and environmentally friendly extraction and post-processing technologies—such as ultrasound-assisted extraction and combined drying methods—should be developed to maximize the retention of bioactive constituents. The ultimate objective is to realize high-value and sustainable utilization of resources. Future work should focus on developing high value-added products derived from its active ingredients—such as terpenoids, polysaccharides, and L-(+)-ergothioneine—for instance, by preparing nanoparticles to enhance oral bioavailability, or by expanding their applications in functional foods, pharmaceuticals, and cosmetics. Through such systematic research, it is anticipated that the challenges of artificial domestication and industrial production of *F. luteovirens* will ultimately be overcome, enabling its transition from a wild resource to an industrially cultivated species, thereby providing a valuable reference for the conservation and utilization of rare edible fungal resources.

## Figures and Tables

**Table 1:** Molecular Omics Study on the Environmental Adaptation and Response of *F. luteovirens*.

Research Dimension	Key Findings	References
Genome and Transcriptome	Genomic annotation of 205 environmental adaptation genes; transcriptome analysis identified 225 Differentially Expressed Genes (DEGs) involved in environmental signal transduction, DNA damage repair and pigment metabolic pathways;	[17]
Functional Genomics	To validate the expression stability of 13 candidate endogenous genes in a wide range of abiotic stresses (salt, drought, oxidation, heat, extreme pH, cadmium stress);	[18]
Population Genomics	Discovery of 15 genotypes based on analysis of rDNA markers (ITS/IGS-1/LSU) to provide a genetic basis for environmental adaptation;	[19]
Transcriptome and Metabolome	The transporter gene *FlMCH5*, which exhibits highly specific overexpression, is a key transport regulatory gene responsible for generating the yellow phenotype;	[20]
Microbial Ecology	Comparison of microbial communities in different regions (IN/ON/OUT) of the fungal ring; Microbial diversity was lower in the ON zone; mycorrhizal promoting bacteria (MHB) such as *Bradyrhizobium* and *Paenibacillus* were more abundant in the ON zone; soil nutrient and physical changes shaped the ON zone community	[21]
Metabolomics	Identification of 5782 metabolites; vanillic acid hypothesized to be a key antioxidant marker; phenylalanine biosynthesis and metabolism as a major differential pathway;	[22]
Transcriptomics	Forty-five core genes for environmental adaptation were identified and classified into signal sensing (28.89%), metabolic reprogramming (26.67%) and stress response (44.44%) categories;	[23,24]

**Table 2:** Biological Functions of Key Bioactive Constituents from *F. luteovirens*.

Metabolite Category	Specific Ingredients	Main Biological Functions	Research Modeling/Methodology	References
Polysaccharide	Extracellular Polysaccharide (FLEP)	Antioxidant; Antitumor; Neuroprotective; Antibacterial and Preservation	In vitro assays: chemical analysis; antitumor (PC9, NCI-H460); neuroprotective (PC12); antibacterial & preservation (shrimp model)	[31,32,36]
	Fruiting body crude Polysaccharides (FLP1, FLPs)	Anti-fatigue, Anti-diabetic Nephropathy, Moisture-absorbing and Moisturizing	Mouse anti-fatigue model, db/db diabetic mouse model, in vitro physicochemical analysis	[33,34,37]
Terpenoid	Melleolides (Capacity)	Genetic basis of terpenoids with antimicrobial and insecticidal activity	Genome mining	[29,38]
	Protoilludane Sesquiterpene Aryl Esters	Antioxidant, Antibacterial	In vitro chemical analysis	[39]
Other high-value compounds	L-(+)-ergothioneine—A rare amino acid	Strong antioxidant, known as the “longevity vitamin”.	HILIC chromatography combined with HPLC-DPPH screening, structure identification	[40]
	Low molecular weight components (<6 kDa)	Anti-tumor: rich in amino acids, nucleosides, terpenes, alkaloids, etc.	Tumor cell lines, hollow fiber membrane separation	[41]
	aqueous extract (FLW)	Anti-type II diabetes, anti-migraine	Type II Diabetes Mellitus Rat Model, Nitroglycerin Induced Migraine Rat Model	[35,42]
	Unsaturated fatty acid	Contains linoleic acid, etc., nutrition and health care.	GC-MS analysis	[43]

## Data Availability

Data sharing is not applicable to this article, as no new data was created or analyzed in this study.
